# Compromised intestinal integrity in older adults during daily activities: a pilot study

**DOI:** 10.1186/s12877-021-02573-4

**Published:** 2021-11-04

**Authors:** Sharon Hendriks, Suzanne C. Stokmans, Matthijs Plas, Wim A. Buurman, Sophie L. W. Spoorenberg, Klaske Wynia, Erik Heineman, Barbara L. van Leeuwen, Jacco J. de Haan

**Affiliations:** 1 Department of Surgery, University Medical Center Groningen, 9713 GZ Groningen, The Netherlands; 2 MHeNs School of Mental Health and Neuroscience, Maastricht University Medical Center, Maastricht, The Netherlands; 3 Department of Health Sciences, Community and Occupational Medicine, University Medical Center Groningen, Groningen, The Netherlands; 4 Department of Neurology, University Medical Center Groningen, University of Groningen, Groningen, The Netherlands; 5 Department of Medical Oncology, University Medical Center Groningen, Groningen, The Netherlands

**Keywords:** Malnutrition, Nutrition, Intestinal integrity, Mesenteric ischemia, Older adults

## Abstract

**Background:**

Malnutrition is a common and significant problem in older adults. Insight into factors underlying malnutrition is needed to develop strategies that can improve the nutritional status. Compromised intestinal integrity caused by gut wall hypoperfusion due to atherosclerosis of the mesenteric arteries in the aging gastrointestinal tract may adversely affect nutrient uptake. The presence of compromised intestinal integrity in older adults is not known. The aim of this study is to provide a proof-of-concept that intestinal integrity is compromised in older adults during daily activities.

**Methods:**

Adults aged ≥75 years living independently without previous gastrointestinal disease or abdominal surgery were asked to complete a standardized walking test and to consume a standardized meal directly afterwards to challenge the mesenteric blood flow. Intestinal fatty acid-binding protein (I-FABP) was measured as a plasma marker of intestinal integrity, in blood samples collected before (baseline) and after the walking test, directly after the meal, and every 15 min thereafter to 75 min postprandially.

**Results:**

Thirty-four participants (median age 81 years; 56% female) were included. Of the participants, 18% were malnourished (PG-SGA score ≥ 4), and 32% were at risk of malnutrition (PG-SGA score, 2 or 3). An I-FABP increase of ≥50% from baseline was considered a meaningful loss of intestinal integrity and was observed in 12 participants (35%; 8 females; median age 80 years). No significant differences were observed in either baseline characteristics, walking test scores, or calorie/macronutrient intake between the groups with and without a ≥ 50% I-FABP peak.

**Conclusion:**

This study is first to indicate that intestinal integrity is compromised during daily activities in a considerable part of older adults living independently.

## Background

Malnutrition is an important condition that negatively affects the health of older adults. It is associated with frailty, osteoporotic fractures, postoperative complications, and an elevated risk of mortality [1–4]. Unfortunately, malnutrition is common in older adults, especially in hospital settings, where up to 40% are reportedly malnourished and almost 50% are at risk [5]. Given the aging population, having a better understanding of malnutrition may help to prevent its occurrence in this vulnerable population and would be of great clinical and economic importance [6].

Malnutrition has a complex multifactorial etiology [7]. Important factors include a decreased sense of smell and taste, compromised gastrointestinal motility, and social factors such as loneliness and depression [8]. Certain diseases and their treatments can also negatively affect nutritional status, of which malignancies are a prime example [9]. Also, chronic inflammation, in the sight of inflammaging, affects the nutritional status in older adults [10, 11]. In addition to these established contributory factors, our overall hypothesis is that a compromised intestinal integrity due to hypoperfusion of the gut wall may impair nutritional absorption and therefore play a role in malnutrition in older adults.

An intact intestinal barrier is essential to protect the bowel from an influx of pathogens, toxins, and other harmful substances, as well as for facilitating nutrient and water transport [12]. A compromised intestinal integrity is therefore detrimental to the health of the individual and is assumed to play a role in inflammatory bowel disease, infectious diarrhea, and extra-intestinal disorders, such as the systemic inflammatory response syndrome, sepsis, and multi-organ failure [13]. Hypoperfusion of the intestinal wall is one of the key factors in compromised intestinal integrity and is thought to result in shortening of bowel villi through abrasion, thereby reducing the absorptive surface area of the gut wall and affecting nutrient absorption [14].

In several conditions, the adaptive capacity of the gut wall is affected, resulting in gut wall hypoxia. For instance, in the event of a reduced circulating volume and shock, blood flow to vital organs such as the brain and kidneys is preserved by severe vasoconstriction in the splanchnic vascular bed [15]. Gut wall hypoxia can also occur during strenuous exercise because splanchnic perfusion decreases to increase muscle perfusion, explaining the high rate of gastrointestinal symptoms in many athletes [16–18]. Another example is non-occlusive chronic mesenteric ischemia, in which the mesenteric vessels have a normal caliber but in which hypoperfusion or hypo-oxygenation are caused by cardiac and pulmonary disease respectively [18–21].

**Fig. 1:**
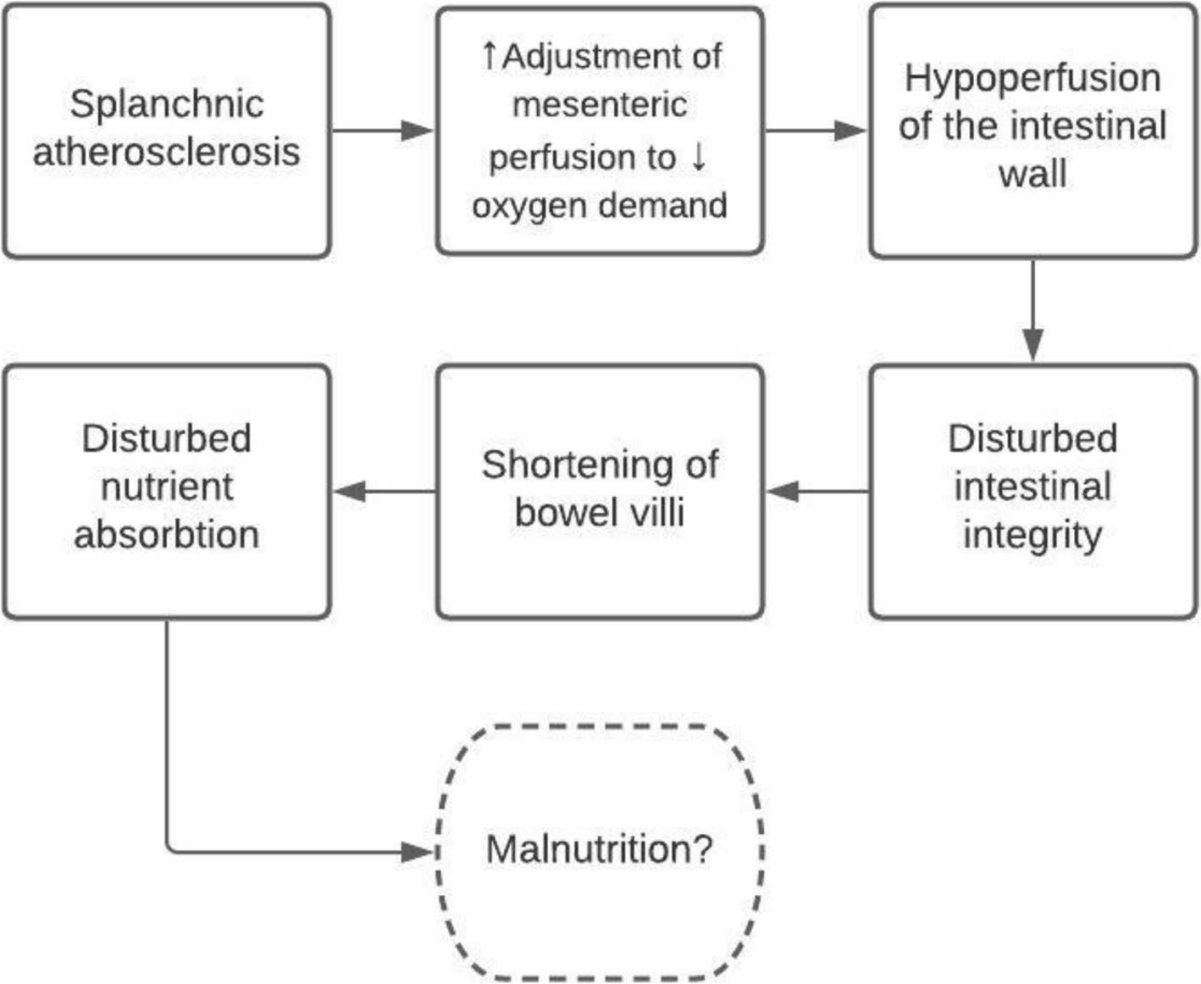
Schematic overview of our overall hypothesis. The normal adjustment of mesenteric perfusion to increased oxygen demand may be diminished in older individuals due to splanchnic atherosclerosis. Activities that increase splanchnic oxygen demands may therefore lead to intestinal wall hypoperfusion, and in turn, cause disturbed intestinal integrity. This process may eventually contribute to disturbed nutrient absorption in older adults if bowel villi shorten adaptively. In this way, atherosclerosis may lead to malnutrition

The aging gastrointestinal tract is prone to hypoperfusion and loss of intestinal function due to multiple factors, including atherosclerosis. Specifically, atherosclerosis of the mesenteric arteries, which is present in 20% of older adults. May impair the capacity to adjust splanchnic perfusion to oxygen demands [22, 23]. During daily activities, this plasticity is needed. Whereas the presence of food in the gastrointestinal tract demands a rapid increase of splanchnic blood flow by 100% to meet the increased oxygen demands of digestion and absorption, physical activity limits gastrointestinal tract perfusion to improve blood flow to the muscles [15, 16]. Activities that require an increase of mesenteric perfusion such as consuming a meal after physical activity, may induce hypoperfusion of the intestinal wall and compromise intestinal integrity (Fig. 1).

Before exploring in depth how compromised intestinal integrity affects nutritional status, nutrient absorption, and digestion in hospitalized older patients, this study was set up to obtain a proof-of-principle that older adults are indeed prone to a compromised intestinal integrity during daily activities.

## Methods

### Ethical statement

The study was approved by the Medical Ethics Committee of the University Medical Center Groningen and registered at clinicaltrials.gov (NCT02831400). This study was conducted according to the latest revision of the Declaration of Helsinki (October 2013, Brazil).

### Aim of the study

To provide a proof-of-principle that older adults are prone to a compromised intestinal integrity during daily activities.

### Participants and procedures

Adults ≥75 years, lived independently, and engaged in the Embrace person-centred integrated care service were eligible. The aim of the Embrace service is to prolong healthy aging by providing person-centered proactive, preventive, and coherent care together with support at home [24]. Recruitment was performed by a single researcher during Embrace community meetings from July to November 2016. Exclusion criteria were previous gastrointestinal resection or history of chronic gastrointestinal inflammation.

After obtaining informed consent, baseline characteristics, such as sex, age, length, weight, allergies, and medication, were recorded at a home visit before the experiment. Daily physical activity level was scored on a binary level based on whether participants engaged in the recommended level of 150 min/week of moderate-intensity activity [25]. Comorbidities were scored using the Charlson Comorbidity Index (CCI) (not licensed) and nutritional status was assessed using the Patient-Generated Subjective Global Assessment tool (PG-SGA) [26, 27].

The experiment was performed on 1 day, starting in the late morning. Participants were not asked to fast and did not receive specific instructions about activities to perform or avoid before attending. During the experiment, participants were free to consume water, coffee and tea.

### Measurement instruments

#### Patient-generated subjective global assessment tool

The PG-SGA has been validated in the Dutch language [27]. The instrument is divided into sections, with the first four scored by the participant: weight history in the past 6 months, 2 months, and 2 weeks; food intake in the past month; symptoms that kept participants from eating in the past 2 weeks; and changes in daily activities in the past month. The final sections concern relevant diseases and their relation to the nutritional requirements and change in metabolic demand (e.g., fever or corticosteroid use), and these are scored by a clinician. Finally, a short physical exam is performed to assess muscle mass, fat reserves, edema, and ascites. An age of ≥65 years is scored as 1 point on the PG-SGA. After completing the assessment, we assigned one of the following three nutritional statuses: well-nourished (0–1 point), moderately nourished or suspected malnourishment (2–3 points), and malnourished (score ≥ 4) [28]. As the PT-SGA is copyrighted, permission to use this tool was requested.

#### Walking test

Participants performed a modified version of the walking test included in the Groningen Fitness Test for the Elderly (not licensed) [29]. In brief, they were instructed to walk clockwise on a 10 × 10 m circuit, with their walking speed dictated by prerecorded tones that gradually reduced the time in which each 10 m was to be walked. The test started at 2.4 km/h and was increased every 3 min to a maximum of 4.5 km/h (paces of 14 s, 12 s, 10 s, and 8 s per 10 m), ending when the participant gave up, was unable to keep up the pace, or successfully completed the test. For safety reasons, participants could use a walker or crutch, and the investigator walked by their side to prevent falls. The maximum test duration was 12 min, and performance was expressed as the total number of 10 m stages completed, giving lowest and highest scores of 0 and 66, respectively.

#### Standardized meal

Directly after completing the walking test, participants consumed a standardized meal consisting of whole wheat macaroni with a tomato sauce, vegetables, and ground beef. One participant had gluten intolerance and was provided with gluten-free pasta. Participants were free to eat at will. Plates were weighed before and after the meals to estimate the calories and macronutrients ingested.

#### Blood samples

**Fig. 2:**
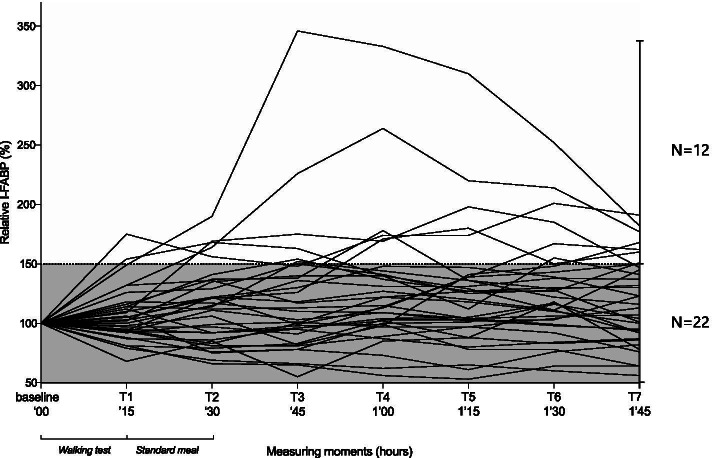
I-FABP concentrations. The relative change in plasma I-FABP (%) compared to baseline (T0 = 100%) at T1 (after walking test), T2 (after meal), and every 15 min postprandially (T3-T7). The dashed line at 150% marks a relative change of ≥50%, defined as a meaningful loss of intestinal integrity. Twelve participants showed I-FABP values ≥50% at least one time point after baseline

An 18-gage venous cannula (Braun, Melsungen, Germany) was placed in the right or left cubital vein and eight blood samples were collected in 10 mL ethylenediaminetetraacetic acid tubes (BD Vacutainer, Becton Dickinson, Helsingborg, Sweden). The first sample was taken after cannula placement to provide a baseline measurement (T0). During the experiment, blood samples were collected directly after the walking test (T1) and then directly after the standard meal (T2) and every 15 min for the first 75 min thereafter (T3-T7) (Fig. 2). The cannula was removed after taking the last blood sample. After centrifugation, blood plasma was stored at − 80 °C until batch analysis.

#### Intestinal fatty acid-binding protein measurement

Plasma I-FABP concentrations were determined by sandwich enzyme-linked immunosorbent assay, according to the manufacturer’s instructions (HyCult Biotech, Uden, the Netherlands), and analyses were performed by HaemoScan (Groningen, the Netherlands). The detection limit of plasma I-FABP is 46 pg/mL. All samples were assessed in duplicate and the mean of the two measures was used in the analysis. However, there is no consensus about the reference range for I-FABP, which is compounded by the fact that studies using I-FABP as a biomarker have been heterogeneous and have used different cut-off values [30, 31]. Based on previous studies of I-FABP dynamics following exercise in relation to protein digestion and absorption, a ≥ 50% increase in plasma I-FABP above baseline was considered to represent a meaningful loss of intestinal integrity [32, 33].

I-FABP is a cytosolic protein that is present in mature enterocytes located on the tips of small bowel villi [13, 33]. These enterocytes are particularly susceptible to hypoperfusion due to the low-flow and low-oxygen environment that results from the distance between the epithelium of the villi and its arterial blood supply. Gut wall hypoxia resulting in epithelial damage is reflected by increased circulatory I-FABP levels, Plasma I-FABP has been shown to be an early and sensitive marker of compromised intestinal integrity [30, 32, 34].

### Statistical analysis

The statistical analyses were performed and reviewed by a biomedical statistician using IBM SPSS Version 22.0 for Windows (IBM Corp., Armonk, NY) and GraphPad Prism version 5.0 for Windows (GraphPad Software, San Diego, CA). Descriptive statistics were used to analyze the course of plasma I-FABP concentrations. Data normality was verified by probability–probability plots. All parametric data are presented as means ± standard error of the mean (SEM), and non-parametric data as medians and range or interquartile range (IQR). Statistical comparison of continuous data between two groups was performed by independent sample *t-*tests for parametric distributions and by a Mann-Whitney *U* tests for non-parametric distributions. Chi-squared tests were used to compare categorical data. A *p*-value < 0.05 was considered statistically significant.

All I-FABP values were transformed to percentages of the baseline value and the primary outcome was set as an increase in the plasma I-FABP level ≥ 50% at any time after baseline. This was expected in 20% of included volunteers given that splanchnic atherosclerosis is reported to be present in 20% of people aged ≥65 years [19, 23, 35]. Conform generally accepted requirements for a pilot study in which there is insufficient data to conduct a power analysis, 30 participants were included [36].

## Results

### Baseline characteristics

In total, 15 men and 19 women (*n* = 34) were included (Table 1). The median age was 81 years (range 75–92 years) and 21 participants (62%) were aged > 80 years. Most (74%; *n* = 25) engaged in at least 150 min/week of moderate physical activity. The median Charlson Comorbidity Index was 3 (IQR 1–3) and the median body mass index (BMI) was 27 kg/m^2^ (range, 24–30), with 22 (65%) participants classified as overweight (BMI > 25 kg/m^2^).

**Table 1:** Baseline characteristics of all participants and by change in I-FABP

	All participants	I-FABP peak <50%	I-FABP peak ≥50%	***p-***value
**Baseline Characteristics**				
Number	34	22 (65)	12 (35)	
Age (years)	81 (79-83)	82 (80-83)	80 (77-82)	0.06
Female sex (%)	20 (59)	12 (55)	8 (67)	0.49
BMI (kg/m²)	26.5 (24-30)	27 (25-31)	26 (24-29)	0.40
BMI >25 kg/m²	22 (65)	15 (68)	7 (58)	0.57
PG-SGA score	1.5 (1-2)	2 (1-3)	1 (1-2)	0.48
PG-SGA ≥4 (malnourished)	6 (18)	5 (23)	1 (8)	0.29
Physical activity ≥150 min/week (%)	25 (74)	14 (64)	11 (92)	0.08
CCI	2 (1-3)	2 (1-3)	1 (0-2)	0.10
**Outcomes**				
Walking test	57 (44-66)	49 (44-66)	66 (39-66)	0.19
Meal (g)				
Total carbohydrates	33 (30-47)	33 (31-45)	34 (30-58)	0.87
Total fat	7 (6-9)	7 (6-9)	7 (5-9)	0.68
Total proteins	16 (13-20)	17 (14-20)	14 (12-22)	0.76
Total Kcal	271 (245-359)	289 (246-350)	249 (224-419)	0.85

Data shown as median (IQR, Inter Quartile Range) or number (percentage). I-FABP: Intestinal fatty acid-binding protein; *BMI* Body Mass Index, *PG-SGA* Patient-Generated Global Assessment, *CCI* Charlson Comorbidity Index

### Nutritional status

The nutritional status among participants were as follows: 17 (50%) were well-nourished (PG-SGA, 0 or 1), 6 (18%) were malnourished (PG-SGA score, ≥ 4), and 11 (32%) were at risk of malnutrition (PG-SGA score, 2 or 3). No participant reported weight loss in the 6 months, 2 months, or 2 weeks prior to enrolment, but 5 (14%) rated their food intake as being less than normal. None were consuming a liquid-only diet or were receiving nutritional supplements, tube feeding, or parental nutrition. In the 2 weeks prior to enrolment, two participants reported problems with eating due to a painful mouth, problems with swallowing, reduced appetite, nausea, and constipation. Interestingly, both participants reported no changes in food intake in the preceding month. Activity levels were normal and without limitations for most participants in the month before enrolment. Three participants reported reduced activity levels: the two above-mentioned individuals, who were still mobile with fairly normal activities, and one participant who was not feeling up to most things and stayed in a bed or chair less than half of the day. All participants scored at least one point on the PG-SGA due to having an age > 65 years. During physical examination, two participants had evidence of mild loss of fat and muscle mass and another two participants had mild ankle edema.

Overall, 15 participants (44%; 7 women and 8 men) fully completed the walking test (660 m in 12 min). The median standardized meal consumed was 285 g (IQR 238–356 g), containing a median of 34 g carbohydrate (IQR 30–47 g), 16 g protein (IQR 12–20 g), 7 g fat (IQR 6–9 g), and 271 kcal (IQR 145–358 kcal).

### Plasma I-FABP levels

A plasma I-FABP increase > 50% above baseline in at least one measurement was observed in 12 (35%) participants (Fig. 2), among whom 8 (67%) were female and the median age was 80 years (IQR 77–82 years). No statistically significant differences were observed in terms of baseline characteristics, walking test scores, or the calories or macronutrients consumed between the 12 participants with a clinically relevant I-FABP increase and the 22 participants with no increase (Table 1).

Two participants showed steeper I-FABP increases than the other participants (Fig. 2). These were both male, aged 79 and 80 years, had BMIs > 25 kg/m^2^, and had a good baseline nutritional status (PG-SGA scores 1 and 2, respectively). Both also achieved the maximum walking test score of 66, and consumed 628 and 242 Kcal, respectively. Apart from the I-FABP increase, no differences were observed between the two participants with pronounced I-FABP increase and the other participants.

**Fig. 3:**
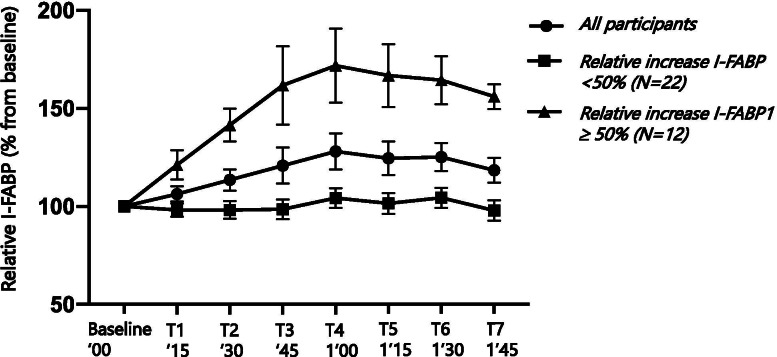
The increases in I-FABP from baseline over time during the study. Relative plasma Intestinal fatty acid-binding protein (I-FABP) values compared to baseline during the experiment are shown in three groups: all participants, those with a relative I-FABP increase < 50% from baseline (*n* = 22), and those with a relative increase ≥50% from baseline (*n* = 12). Data are expressed in mean ± standard error of the mean (SEM)

Most I-FABP peaks occurred between 30 and 60 min postprandially (T2-T4, Fig. 3). Six participants (18%) did not show an I-FABP increase. After the baseline measurement, I-FABP serum levels in these participants decreased and did not exceed the baseline concentration in any subsequent blood sample.

## Discussion

This proof-of-concept study showed that I-FABP plasma levels increase at least 50% above baseline when a walking test is followed by a meal in 35% of a group of independently living older adults. These findings indicate that intestinal integrity is disturbed to some extent during daily life activities that challenge the mesenteric blood flow. As such, the study can be used to guide future research into the clinical relevance of compromised intestinal integrity and the pathogenesis of malnutrition in older adults.

The overall hypothesis is that loss of the plasticity that is needed to adjust splanchnic perfusion, to the oxygen needs of the gut wall in older adults contributes to a compromised intestinal integrity and nutritional malabsorption, even under normal conditions [13, 33]. Splanchnic atherosclerosis is a potential key factor, effectively reducing vascular plasticity and compensatory mechanisms. In turn, this may increase the arterial blood flow and lead to transiently inadequate intestinal perfusion either postprandially or during exercise [16, 17, 23].

Even short periods of asymptomatic intestinal hypoperfusion can induce tissue damage and contribute to disturbed bowel integrity [14, 30]. In healthy young men performing resistance-type exercise, protein malabsorption has been found to occur in the post-exercise recovery period, with the extent of malabsorption related to the extent of intestinal compromise, as measured by I-FABP levels [37]. The post-exercise recovery period for blood flow, and thus the period of protein malabsorption, was relatively short in that study. By contrast, we found that older adults with an I-FABP increase ≥50% did not show a return to baseline values by the end of the experiment, potentially indicating that intestinal integrity had not yet been restored. It is therefore plausible that impaired intestinal function might persist for longer in older adults after provocation, especially if several episodes of intestinal compromise occur in rapid succession without adequate time for the intestinal wall to recover. When hypoperfusion occurs often, the intestinal surface area may diminish due to a shortening of bowel villi, leading to a decreased nutrient absorption capacity of the gut wall [23, 30].

The current study was specifically designed to create a challenge to the mesenteric blood flow by exposing older adults to physical activity followed immediately by a meal. This provocation led to a relevant increase in I-FABP levels in 35% of participants. Two participants had an increase ≥50% of the baseline I-FABP immediately after the walking test (before the meal), raising the question whether physical activity alone might be sufficient to cause disturbed intestinal integrity in older adults. Unfortunately, participants were not asked about gastrointestinal symptoms during the experiment and the caliber of the mesenteric arteries was not measured, so whether the 12 participants with increased I-FABP levels had mesenteric artery stenosis, atherosclerosis, or chronic intestinal ischemia is unknown [19]. Future research should distinguish if other important factors contribute to the disturbed intestinal integrity.

Regular exercise may condition the colon to tolerate reduced flow by making vascular adaptations to the intestinal wall that make it less prone to damage by hypoperfusion. This principle is used in prehabilitation programs in which patients planned for colon resection are physically trained to generate optimal intestinal blood flow and which is thought to result in better anastomotic healing [38]. Given that almost three-quarters of participants engaged in at least 150 min/week of physical activity, we conclude that the present study was performed in active older adults. In line, many participants also achieved the maximum score in the walking test. It is possible that inactive or frail older adults show a more pronounced loss of intestinal integrity after physical activity than the relatively healthy and active participants included in this pilot study.

Comorbidities can negatively influence intestinal integrity. In a study comparing healthy controls to patients with moderate chronic obstructive pulmonary disease (COPD), it was shown that I-FABP levels were significantly higher in the latter when performing activities of daily living [20]. The general oxygen deficit in patients with COPD was suggested to cause hypoperfusion of the intestinal wall. Similarly, patients with chronic heart failure, a condition in which a systemic oxygen deficit is caused by decreased cardiac output, have been shown to have increased gastrointestinal permeability compared with healthy controls [21]. Given that most comorbidities are age-related, it is possible that a systemic oxygen deficit negatively affected intestinal integrity in our cohort when the splanchnic blood flow is challenged. Furthermore, possible vasoconstrictor effects of smoking on the superior mesenteric artery are described, possibly producing unwanted results in patients who are chronic smokers, such as stenotic lesions in this artery and chronic intestinal ischemia [39, 40]. To our knowledge, the effects of smoking on I-FABP levels have not been described. In this cohort, smoking could influence splanchnic blood flow and intestinal integrity but was not asked for. In future studies, it would be useful to perform studies like these in larger cohorts to be able to perform multifactorial analysis.

In addition to comorbidities, age related changes of the gastrointestinal tract can negatively influence intestinal integrity. Age effects all functions of the gastrointestinal tract: motility, enzyme and hormone secretion, digestion and absorption [41]. For example, small intestinal bacterial overgrowth is common in older adults. This condition is associated with chronic diarrhea, malabsorption, weight loss and secondary nutritional deficiencies [42]. Also, Shintouo et al. found that age related changes in gut microbiota are associated with inflammaging, a chronic low-grade inflammation that develops with advanced age that affects nutritional status in older adults [10, 11, 43]. Taken together, age related changes are important in the understanding of the development of malnutrition and should be taken into account in future studies.

This study was not designed to investigate the mechanisms underlying compromised intestinal integrity. None of the included baseline characteristics were significantly associated with an I-FABP increase. Therefore, risk factors for the loss of intestinal integrity during provocation of the splanchnic vasculature need to be clarified. Future research on this topic should include assessment of splanchnic atherosclerosis. Another issue is that participants were not asked to perform or avoid any activities before the experiment, so we cannot exclude that some may have undertaken physical activity prior to the test, resulting in an altered I-FABP at baseline. Noteworthy, I-FABP levels decreased in six participants during the experiment. For future studies, we advocate a more controlled research environment where participants are fasted and have a few hours of rest prior to the experiment, and multiple baseline measurements are taken.

A potential limitation of this study is the unknown use of over-the-counter nonsteroidal anti-inflammatory drugs (NSAIDs), which is important given that high levels of I-FABP occur during exercise in healthy individuals using NSAIDs [44]. However, we did record prescribed medication use, and a sub-analysis showed not only that the seven participants who used a prescribed NSAID (acetylsalicylic acid) had comparable I-FABP levels but also that there was no difference in NSAID use between the groups with and without a ≥ 50% increase in I-FABP levels. Further, intestinal integrity was only assessed using I-FABP, and although this is an early and sensitive marker of compromised intestinal integrity, the gold standard for assessing gastrointestinal mucosal perfusion is tonometry [45]. In future studies renal function should be determined as plasma I-FABP concentrations are elevated in patients with diminished kidney function [46]. Finally, to determine if the compromised intestinal integrity found in this study is specific for older adults, the experiment should be repeated in younger adults.

Based on this proof-of-concept study, further studies are being set up to study intestinal integrity in older and younger adults when challenging splanchnic blood flow in daily activities and in clinical settings. Next to I-FABP, gut wall permeability, inflammatory parameters, nutrient absorption and mesenteric blood flow will be assessed. A better understanding of the role of compromised intestinal integrity in the development of malnutrition may also provide novel directions to prevent or treat malnutrition, for example by addressing the effects of timing of consuming meals and performing exercise. Furthermore, the determination of intestinal integrity is a potential tool to aid patients and health care professionals in making treatment decisions for example regarding major surgery and or chemotherapy.

## Conclusions

This proof-of-concept study shows that intestinal integrity is compromised in a considerable part of community-dwelling older adults following daily life activities. These results can be used in future studies that investigate more in-depth the relation between food intake, exercise, intestinal integrity and nutritional status in the elderly population.

## Data Availability

The data that support the findings of this study are available on request from the corresponding author.

## Abbreviations

I-FABP: Intestinal Fatty Acid Binding Protein
PG-SGA: Patient-Generated Subjective Global Assessment
CCI: Charlson Comorbidity index
SEM: Standard Error of Mean
IQR: Interquartile Range
BMI: Body Mass Index
COPD: Chronic Obstructive Pulmonary Disease
NSAID: Non-Steroid Anti-Inflammatory Drug
